# The development of the 5Cs of positive youth development in a school year: A 3-wave longitudinal study of Slovenian youth during the COVID-19 pandemic

**DOI:** 10.3389/fpsyg.2023.982856

**Published:** 2023-04-05

**Authors:** Ana Kozina, Nora Wiium

**Affiliations:** ^1^The Educational Research Institute, Ljubljana, Slovenia; ^2^Department of Psychosocial Science, University of Bergen, Bergen, Norway

**Keywords:** 5Cs of PYD, school year, age, gender, school level, COVID-19 pandemic, Slovenia, longitudinal analysis

## Abstract

The paper analyses the longitudinal pathways for the 5Cs of positive youth development outcomes (Competence, Confidence, Character, Caring, Connection) in a school context during the COVID-19 pandemic. The theoretical framework for the paper is provided by the Positive youth development (PYD) perspective, which focuses on the importance of the interplay between individual characteristics and contexts. In the period of adolescence, school and its characteristics are one of the most influential contexts for the promotion of positive youth development. Therefore, the paper focused on the changes in the 5Cs in one school year using the methodology of longitudinal research (latent growth modeling) with three measurement points (beginning of the school year, middle of the school year, and the end of the school year). We used a sample of Slovenian youth (*N* = 1241 participants; 59,5% female; *M*_age_ = 15.35, *SD* = 1.21) who participated in the PYD-SI-MODEL study and responded to the PYD questionnaire during the current COVID-19 pandemic. In addition, we tested whether the development of the 5Cs in a school year differed by gender, age, and school level (lower-secondary, upper-secondary). The results indicated a significant decrease in Connection, Caring, and Character from the beginning to the end of the school year and an increase in Competence and Confidence in the same period. Significant intercept-slope interactions were detected in Confidence and Connection indicating that their higher initial level is associated with a more stable trajectory throughout the school year. In addition, we found that gender and school level play a significant role in several of the pathways while age was not a significant covariate with any of the Cs. The study is the first to test 5Cs pathways in one school year, using Slovenian data. In addition to the important role of contexts on positive developmental outcomes, the study highlights the significance of 5Cs promotion in pandemic times as much as in more stable times.

## Introduction

The school context significantly shapes the development of youth (Wang and Fredricks, 2014). It is influenced by a wide range of factors from macro (e.g., national, regional, or local school system) to meso (e.g., home and school context interaction) and micro (e.g., relationships in the classroom) level. The present paper focuses on how positive youth development is shaped in the context of macro (e.g., COVID-19 pandemic), meso (progression of the school year, interaction between home and school) and micro level influences (e.g., relationships in the classroom). In the last years, the COVID-19 pandemic and restrictions imposed to prevent the spread of the virus (e.g., school lockdowns) were one of the most influential macro-level influences affecting the school system. As such the COVID-19 pandemic affected also meso-and micro-level influences.

In addition to the COVID-19 context, it is also important to note that the school context and its complexities change significantly throughout the normative school year and these changes can influence the positive development of youth. On one hand, these changes are a reflection of the learning and teaching process in one school year, from its introductions and “getting to know each other” at the beginning of the school year to the “stress of final exams” at the end of the school year. On the other hand, changes at school include intervention and prevention programs taking place in the school year. Research suggests that youth who participate in school-based positive youth development activities also report an increased sense of school belonging (Herrera et al., 2011), leading to less probability of school drop-out (Lerner et al., 2005). School is especially influential in the period of adolescence due to the associated development that is characterized by building a sense of identity through group membership (Steinberg, 2004) alongside a burst in neurological development under the strong influence of environmental factors, such as those related to the school context (Patel, 2013).

In the present paper, we investigate indicators of positive youth developmental outcomes throughout one school year using the theoretical framework of Positive Youth Development (PYD) (Lerner, 2007) and having in mind the changing school year context as well as the COVID-19 context and the way it might have shaped positive youth development in Slovenia in the last years. As the school year is a time-frame that is more influential for the lives of youth compared to the calendar year, we have used this time-frame. We have followed the youth from the beginning to the end of one school year. This way we can observe the overlap of the processes marked by changes in the school year as well as changes in the pandemic progression and the restrictions associated with it.

### The PYD framework and the 5Cs

The PYD framework is based on Relational Developmental System Theory, which focuses on the importance of the interplay between individual characteristics and their contexts (Lerner, 2007, 2017). Developmental System Theory argues that young people should be studied as a product of a two-way interaction between the individual and his or her environment. The basic idea is that youth will develop positively when their strengths (internal developmental assets) are aligned with the resources in their ecology (external developmental assets). Thus, positive outcomes will be more probable and risky behaviors (e.g., early school leaving) less frequent. Lerner (2007) operationalized positive outcomes as 5Cs: Competence, Confidence, Character, Connection, and Caring. Confidence is defined as an internal sense of positive self-worth, self-efficacy, appearance, and positive identity. Competence is a positive view of one’s actions in domain-specific areas (social, sports, and academic competencies). Connection represents all the positive reciprocal bonds of an adolescent with significant others and institutions (peers, family, school, community). Character is defined as a possession of standards for correct behavior with respect to societal and cultural norms (personal and diversity values, social conscience, conduct behavior). A sense of sympathy and empathy for others is reflected in Caring. Together, the 5Cs are viewed as an antecedent of a sixth C – contribution to self, family, community, and institutions in civil society (Lerner, 2007).

The trajectories of the 5Cs are largely stable in time, meaning that a youth on a low Cs trajectory is most likely to stay on that trajectory throughout adolescence as shown in a US-based 4-H longitudinal sequential design study (Lerner et al., 2005). The longitudinal study, that included more than 7,000 adolescents from USA, aimed to assess the effectiveness of its youth development programs and identified four pathways that indicate relative stability in PYD indicators across adolescence ranging from optimal (high Cs) to problematic (low Cs) and gradations in-between. The first optimal trajectory consisted of 25.1% of their youth sample, the second, 42.3%, the third, 26.8% and the fourth problematic trajectory consisted of 5.7% of their youth sample. More females were represented in the first two, more optimal trajectories, and more males in the last two, more problematic trajectories. This goes well with the cross-sectional data from the first wave of the same study involving 1700 5*^th^* graders showing that females report higher overall PYD scores than males (Lerner et al., 2005; Phelps et al., 2009).

Moreover, specific gender differences across the 5Cs have been observed, such that females reported more Connection, Character, and Caring, while males presented higher scores on Confidence in several studies; for example, a cross-sectional study investigating the mediating role of the 5Cs on the relationship between school empowerment and school satisfaction together with gender effects in a sample of Norwegian upper-secondary students (Årdal et al., 2018); a cross-sectional study in Irish early and late adolescents analyzing measurement properties of the 5Cs model (Conway et al., 2015), and a cross sectional study investigating gender differences in a Spanish sample of late adolescent and emerging adults (Gomez-Baya et al., 2019). The last study (Gomez-Baya et al., 2019), identified additional significant differences in Competence, with males scoring higher compared to females.

More ambiguity has been found in age differences in the 5Cs. For instance, Gomez-Baya et al. (2019) did not detect any age differences across the 5Cs, whereas Conway et al. (2015) reported that younger adolescents showed higher Caring, Character, and Connection than older adolescents but found no age differences for Confidence and Competence. Bowers et al. (2010) on the contrary found significantly higher levels of Competence, Confidence, and Connection in younger adolescents compared to older adolescents but lower Caring and Character using data from 8th to 10th graders from the 4-H study. Additionally, an earlier cross-sectional study (Kozina et al., 2019) highlighted the importance of variations in the school context (e.g., school type) in addition to gender when analyzing the associations between the 5Cs and outcomes, specifically academic achievement, in a large Slovene youth sample. Besides gender and age, school type (lower versus upper secondary) is the focus of the present study.

### PYD and the school context

As noted above, school as a context, changes throughout the school year. For instance, the beginning of the school year is characterized by new classmates, new teachers, new subjects, and fewer learning requirements, especially if starting a new school. The middle of the school year is more stable and with a strong focus on learning and teaching, while the end of the school year can be either socially more stable, for example, stable peer groups, or a time of increased stress with final exams.

Concerning how the 5Cs of PYD may be influenced in the school context, research (Escribano et al., 2021) shows that the number of peer connections increases from the beginning to the end of the school year. More specifically, when early adolescents arrive at the upper secondary school, their relationships are at the beginning of the school year intense but with a smaller, limited number of peers, and by the end of their first year in upper-secondary school the structure had changed from fewer intense peer relationships to more peer relationships (Escribano et al., 2021).

Being more connected throughout the school year could also be a result of an increase in empathy and caring, which play a key role in the development of social understanding and positive social behaviors (Schultz et al., 2003). However, it is assumed that by middle adolescence the cognitive and emotional components that support empathy are fully matured or at least developed to a stable status and therefore an increase would not be expected in one school year (Barr and Higgins-D’Alessandro, 2009). Similarly, character and moral reasoning in a form of self-reflective perspective-taking and other-oriented judgments tend to emerge in late childhood and increase through adolescence (Schultz et al., 2003). By late adolescence, an individual has gained the ability to consider multiple perspectives, feel concerned, and incorporate them when analyzing and acting upon situations (Eisenberg, 1990). Academic competence and confidence are more dependent on situational factors, such as changes in a school year due to gaining more subject knowledge. Thus, one would expect improvement in one school year.

### Slovenian school context

Slovenia has a documented high-quality education system with results above the OECD average (Organization for Economic Co-operation and Development [OECD], 2019). The country’s education system is structurally organized into preschool care (11 months to 6 years), basic school (6 years to 15 years of age), upper-secondary education (from 15 years on), and tertiary education. The basic school comprises primary and lower secondary education. In line with the Constitution of the Republic of Slovenia, basic school education is compulsory and funded by public revenues. Children must enroll in first grade at the age of six. Schools implement the single-structure curriculum over the course of nine years, and pupils typically complete basic school education at the age of 15. Upper secondary education consists of 2- to 5-year non-compulsory school for students who have completed compulsory basic education. Upper secondary education can take the form of a 4-year gymnasium program, 4-year upper secondary technical education, 3-year upper secondary vocational education, or a 2-year short, upper secondary vocational education. There are some significant structural and organizational differences between lower-secondary level and upper-secondary level in Slovenia. The lower-secondary level is compulsory, located within proximity of ones’ home, and students stay in the same class with the same classmates for the 9-year duration. Alternatively, the upper-secondary level is non-compulsory, not the same for all, sometimes located further away from home, and the students are in the same class with the same classmates for the duration of 2-5 years, depending on the form of upper-secondary education (Euridyce, 2022).

In the 2020/2021 school year, 193,158 students were attending basic education and 73,854 students were in upper secondary education in Slovenia (SURS, 2021). Given that almost all (98%) of adolescents in Slovenia attend upper secondary school, schools have enormous potential to foster positive youth development in Slovenian youth. Unfortunately, in Slovenia, PYD-related intervention has not gained systematic support even though a growing research body (Kozina et al., 2019, 2021) supports its implementation.

### The COVID-19 pandemic

In addition to the changes in the school year, during the past two years, the school context has been significantly interrupted by the COVID-19 pandemic. COVID-19 has brought tremendous challenges to individuals, families, societies, and the world. Research shows that youth were more at risk compared to other age groups (Power et al., 2020), an assertion that is also true in Slovenia (Avsec et al., 2020). In addition to health-related threats and social distancing restrictions, adolescents faced challenges associated with school lockdown, online teaching and learning, together with a reduction in teacher guidance and peer interaction, which are important sources of support for adolescents. For instance, the change to online schooling triggered extra worries about schooling and peer relationships (Ellis et al., 2020) as well as depression, and loneliness due to less contact with classmates (Esposito et al., 2021).

There are several studies (e.g., Chen et al., 2021; Ravens-Sieberer et al., 2021) focusing on the negative effects of COVID-19 on mental health in the youth population and less on the protective factors. Shek et al. (2021) argued that it is important to understand risk factors for youth development, such as the perceived threat of COVID-19 although it is equally important to focus on protective factors. In his study, Shek et al. (2021) showed the protective effect of positive youth development attributes in reducing the negative influence of traumatic situations, such as COVID-19 on adolescent mental health. Moreover, research has been mostly conducted using late adolescents, especially university students (e.g., Cohen et al., 2020; Li et al., 2020) while limited research has been conducted on early and middle adolescents (e.g., Duan et al., 2020; Liang et al., 2020), a sub-population that may face even more difficulties while still developing their coping strategies.

### Slovenian school context in the COVID-pandemic

Like other European nations, Slovenia declared a COVID-19 epidemic on the 12th of March 2020. There was therefore a lockdown between the 16th of March and 18th of May for kindergartens and the first three grades of basic schools and until the 25th of May for the rest of the students in basic and upper-secondary schools. In autumn 2020, the second lockdown followed with the closing of all schools from the 23rd of October 2020, until the 15th of February 2021 for basic schools and final grades of upper-secondary school. The rest of the grades in upper-secondary schools returned to school on the 7th of March with additional restrictions (e.g., every other week homeschooling). After returning to school, social distancing restrictions measures were put in place, for example, contact was limited to one’s own class, while social distancing and wearing of face masks were enforced. In the present study, we have followed students from the first school lockdown in October 2020 until May 2021 when they returned to school.

### The present study

In the present study, we focus on the development of the 5Cs of PYD in one school year. Despite earlier findings on the stability of the 5Cs (Lerner et al., 2005), due to the COVID-19 pandemic and associated restrictions, such as the social distancing restrictions and online schooling, we do not expect as much stability as has been observed in previous research. We hypothesize therefore that.

• Connection would decrease from the beginning to the end of the school year as a result of school closures and lockdown taking place for the most part of the school year as well as social distancing and other restrictions following the re-opening of school. Our expectation is supported by research (Esposito et al., 2021) that shows higher levels of loneliness during the COVID-19 pandemic.
• Caring would decrease from the beginning to the end of the school year due to the lack of social connections (e.g., home schooling, social distancing restrictions) and limitation of contact to only close family members. This is because isolation and the lack of social interactions with peers and friends have been found to be negatively associated with empathy and compassion (Silke et al., 2018).
• Competence and Confidence would decrease in light of the ongoing COVID-19 restrictions, for instance, academic competence due to difficulties adapting to online schooling (e.g., technical equipment, digital competencies, inadequate learning environment), sports competencies due to limited sports activities (e.g., in a closed setting, group sports), and social competencies due to aforementioned limited social interactions.
• finally, we hypothesize that Character would not be that much affected by the school year and the COVID-19 restrictions, thus, Character would remain stable from the beginning to the end of the school year.

In addition, we investigate whether the initial level and rate of change of the 5Cs, are associated with age, gender, and school level (lower-secondary, upper-secondary). Due to previous research (Gomez-Baya et al., 2019; Kozina et al., 2019) we expect a significant association of the 5Cs with gender and school level.

To our knowledge, the present study is the first of its kind to analyze longitudinal 5Cs trajectories in a non-US sample while also considering the context of the COVID-19 pandemic. As most of the school year was influenced by remote schooling and youth not being in school, we believe that the study brings important theoretical insights into how the withdrawal of school, as a significant context, can shape the development of youth. Additionally, insight into the influence on youth development can have important practical implication for fostering PYD interventions in normative and non-normative school years, such as, the COVID-19 pandemic. Slovenia had one of the longest school closures in the second wave of the pandemic (2020–2021) when compared to other EU countries (United Nations Educational, Scientific and Cultural Organization [UNESCO], 2021) and can therefore be used as a case study.

## Materials and methods

### Participants

In line with the research aims, all lower and upper secondary school types in Slovenia were sampled taking into consideration, the proportion of students that attended each type of school in Slovenia. Furthermore, all lower and upper secondary schools were divided into two groups according to the number of additional hours of Slovenian language that was offered to migrant students. Lower and upper secondary schools with the highest number of additional hours of Slovenian language for migrant students were invited to participate in the study. In the meantime, another group of lower and upper secondary schools that did not have any additional hours of Slovenian language for migrant students was randomly sampled and invited to participate in the study. When schools agreed to participate, further sampling of the classes of upper secondary schools was carried out.

The initial sample of the present study (i.e., the baseline or T1) included 1,984 participants from Slovenia (57.4% female, 42.5% male, 0.1% non-binary), aged 13 to 19 years (*M* = 15.34; *SD* = 1.19). The majority of participants were attending one of 20 upper-secondary schools (1,406 students; 70.8%; 57.8% females). The age of these students varied from 14 to 19 years (*M* = 15.91; *SD* = 0.91). The rest of the participants were attending one of 21 lower secondary schools (578 students; 29.7%) and were between 13 and 16 years (*M* = 13.96; *SD* = 0.38). Most of them were females (56.3%). In the study, we have used data from participants that were involved in all three waves of data collection, that is 1241 participants (59.5% female, 40.3% male and 0.2% non-binary), between ages 13 and 19 years (*M* = 15.33; *SD* = 1.20). For the final sample, two thirds of the students attended upper-secondary school (69.8%) and one third attended lower-secondary school (30.2%). Due to small numbers, non-binary persons (2 persons) in relation to gender were excluded from the analyses.

### Instruments

In the study, we used the short form of the PYD questionnaire to measure the 5Cs alongside a set of demographics that were included in a survey for the study *Positive Youth Development in Slovenia: Developmental Pathways in the Context of Migration.*

The short form of the PYD questionnaire (Geldhof et al., 2014) consists of 34 items answered on a 5 point Likert scale (with responses ranging from, for example, 1 = strongly disagree to 5 = strongly agree). Sample items for the 5Cs include: Competence (“I do very well in my class work at school”), Confidence (“All in all, I am glad I am me”), Caring (“When I see another person who is hurt or upset, I feel sorry for them”), Character (“I hardly ever do things I know I shouldn’t do”), and Connection (“My friends care about me”). The PYD questionnaire has proven to be psychometrically valid in the sample used in this study with reliability coefficients at T1: 0.705 (Competence); 0.921 (Confidence); 0.718 (Character); 0.863 (Caring); 0.796 (Connection); at T2: 0.756 (Competence); 0.922 (Confidence); 0.745 (Character); 0.893 (Caring); 0.822 (Connection); and at T3: 0.757 (Competence); 0.926 (Confidence); 0.764 (Character); 0.906 (Caring); 0.835 (Connection). CFA (Confirmatory Factor Analysis) confirmed an adequate fit of the 5-factor structure at T1: X^2^ (507) = 3595.25, *p* < 0.001, RMSEA (Root Mean Square Error of Approximation) = 0.055, 90% CI [0.054, 0.057], CFI (Comparative Fit Index) = 0.905; SRMR (Standardized Root Mean Squared Residual) = 0.066 (Pivec, 2021).

Gender (Open ended question *What is your gender* was recoded into 1 = female, 2 = male, 3 = other), Age (Open ended question: What is your age) and school level (lower-secondary school, upper-secondary school) were included in a set of demographic variables.

### Procedure

The present study was approved by the Committee for Ethical Research at the Faculty of Arts of the University of Maribor. After obtaining informed consent from their parents, the students responded either on paper or online due to the COVID-19 situation. Participants were not rewarded for their participation. The data collection time was not limited, and they were supervised by the school coordinator (teacher or school counselor) who answered any questions whenever necessary. The first data collection (i.e., T1) took place during the second wave of the COVID-19 pandemic in Slovenia (between October and December 2020). As part of the subsequent restrictions, there was a school lockdown with remote schooling that began on the 19*^th^* of October. T2 data collection took place between January and March 2021 (with school lockdown still ongoing). The lower-secondary school students went back to school after the 15*^th^* of February while the upper-secondary school students went back after the 8*^th^* of March. T3 data collection took place between May and June 2021 with all the students back in school.

### Data analysis

After examining the descriptive statistics, correlations, and reliabilities using IBM SPSS Statistics 28, we estimated measurement invariance (i.e., construct, metric, and scalar invariance) for the data collected at the three-time points for each of the 5Cs. Measurement invariance assesses the (psychometric) equivalence of a construct across groups or measurement occasions and demonstrates that a construct has the same meaning across groups or repeated measurements. Establishing measurement invariance, especially scalar, helps to make meaningful comparisons between the groups. If configural measurement invariance was not achieved modification indices were used. A change in CFI (equal or less than 0.01) was used as an indicator of measurement invariance since chi-square difference tests are dependent on the sample size (Cheung and Rensvold, 2002). However, Rutkowski and Svetina (2014) also argue that fit indices change in CFI of 0.02 and RMSEA of 0.03 were appropriate for tests of metric invariance with large group sizes, as in our case. As our data were nested, we calculated intraclass correlation coefficients (ICCs) to examine the shares of variance at each level. The ICCs ranged between 0.003 and 0.047 for schools and between 0.009 and 0.007 for school classes. As the ICCs were lower or at the suggested cutoff of 0.10 (Peugh, 2010) and the aims of the present study concerned individual characteristics, we decided to perform the data analyses at the individual level. We then employed the latent growth curve models (LGCM) to examine the longitudinal change over time using Mplus (Version 8.6; Muthén and Muthén, 1998–2021). The latent growth curve model is a useful tool in analyzing longitudinal data, allowing tracking of the trajectories and changes over time. Furthermore, the analytical procedure allows researchers to include variables to predict parameters of the trajectories (Bollen and Curran, 2006).

A robust maximum likelihood (MLR) algorithm was used to handle missing data and assess parameters in the model. With the maximum likelihood algorithm, the estimates of parameters and their standard errors are based on all available data (Peugh and Enders, 2004). First, we estimated the unconditional LGCMs, where the latent factors (intercept and slope; the parameters describing the growth curve) were created for five observed repeated measures (i.e., Competence, Confidence, Character, Caring, and Connection). This allowed us to examine the intra-individual change over time. The intercepts were constrained to be equal (i.e., they were fixed to 1) and values assigned to the factor loadings of the slope reflected the data collection time intervals (i.e., each value represented three months). In the second step, conditional LGCMs were examined by adding covariates into the model (gender, age, school type) at the individual level (estimating the effects of the covariates on the latent growth parameters). A direct effect of the covariates enabled us to examine whether the covariates explained (some of) the inter-individual differences in the growth curves (Stoel et al., 2004). The following cut-off values were applied for adequate fit: CFI > 0.90, RMSEA < 0.08 and the SRMR < 0.08 (Hair et al., 1998).

## Results

After reporting measurement invariance and descriptive statistics, we present the unconditional and conditional LGC models for the 5Cs.

### Measurement invariance

For Competence, Confidence, Caring, and Character, the configural invariance model indicated adequate fit, meaning that similar patterns of observed and latent constructs across time points were achieved. A slightly worse fit was established for Connection, but still adequate (indicated by an RMSEA below 0.10). A well-fitting configural invariance model suggests that additional measurement invariance tests may proceed. For Competence and Confidence, fit indices of the metric invariance model, in which factor loadings of the items were constrained to be equal across time points, showed adequate fit, and the change in CFI was equal to or below 0.01. As for Caring, Character, and Connection, the change in CFI was greater than 0.01 but below or equal to 0.02. According to Rutkowski and Svetina (2014), fit indices change of 0.02 can be considered. For the scalar invariance model, in which the intercepts of the items were fixed to be equal across time points, Competence, Confidence, Character, and Connection showed adequate or good model fit, and the change in CFI was equal to or below 0.01. The CFI difference in Caring was 0.02 (see Table 1).

**TABLE 1:** Measurement invariance models and goodness-of-fit indexes of the 5Cs across three time points.

Model	Model fit indices			
	χ^2^ (df)	RMSEA	90% CI RMSEA	CFI
**Connection**				
Configural invariance	13,747.440 (276)	0.082	0.078–0.085	0.870
Metric invariance	13,747.440 (276)	0.074	0.070–0.077	0.889
Scalar invariance	13,747.440 (276)	0.073	0.070–0.076	0.883
**Caring**				
Configural invariance	11,373.993 (153)	0.059	0.054–0.064	0.957
Metric invariance	11,373.993 (153)	0.044	0.039–0.048	0.974
Scalar invariance	11,373.993 (153)	0.056	0.052–0.060	0.954
**Competence**				
Configural invariance	10,658.723 (153)	0.081	0.076–0.086	0.920
Metric invariance	10,658.723 (153)	0.071	0.067–0.076	0.932
Scalar invariance	10,658.723 (153)	0.074	0.070–0.078	0.919
**Confidence**				
Configural invariance	15,097.295 (153)	0.047	0.042–0.051	0.981
Metric invariance	15,097.295 (153)	0.046	0.041–0.051	0.980
Scalar invariance	15,097.295 (153)	0.050	0.046–0.055	0.973
**Character**				
Configural invariance	7,366.373 (153)	0.064	0.060–0.069	0.924
Metric invariance	7,366.373 (153)	0.053	0.048–0.057	0.945
Scalar invariance	7,366.373 (153)	0.054	0.050–0.059	0.936

χ^2^, Chi-square; df, degrees of freedom; CFI, Comparative Fit Index; RMSEA, root mean square error of approximation; CI, confidence interval. Two items targeting conduct behavior in Character were deleted from the further analyses based on their lower loading onto Character in measurement invariance analyses (see Kabir and Wiium, 2021). Lower loadings for these two items were detected also in the scale development study (Geldhof et al., 2014). Reliability coefficients with 6 items are at T1 (0.729), at T2 (0.754), and at T3 (0.776).

### Descriptive results

Means and standard deviations of the 5Cs at the three time-points are presented in Table 2. Skewness and kurtosis values, at T1 skewness varying between −1.634 and 0.027 and kurtosis between −0.875 and 3.177, at T2 skewness varying between −1.471 and 0.007 and kurtosis between −0.884 and 2.481, at T3 skewness varying between −1.344 and −0.034 and kurtosis between −0.899 and 2.048, were considered acceptable for all included variables before computing the 5Cs composite scores.

**TABLE 2:** Means, standard deviation of the 5Cs for the three time points.

	T1		T2		T3	
	*M*	*SD*	*M*	*SD*	*M*	*SD*
Connection	3.732	0.640	3.442	5.084	3.358	5.859
Caring	3.931	3.016	3.748	4.203	3.528	5.883
Competence	3.384	0.655	3.349	0.703	3.419	0.704
Confidence	3.527	0.927	3.551	0.904	3.598	0.899
Character	3.951	0.598	3.931	0.611	3.911	0.637

We observed a decrease in Competence, especially from T1 to T2 and a decrease in Caring and Connection from T1 to T3. Confidence and Characters were relatively stable across the three time points.

### Latent growth curve models

#### Unconditional LGC models

Unconditional LGCMs (measurement model, without covariates) were used to calculate the intra-individual differences in the growth curve of the 5Cs over three time points (within-person model) in one school year.

Table 3 shows that all unconditional LGCMs have a good fit with the data. In all models, the means and the variances of the intercept (the average initial levels and the inter-individual differences in the initial levels of the 5Cs) were statistically significant. The average intra-individual change (the mean of the slope) was statistically significant for Competence, Confidence, Caring and Character, with negative values indicating a significant rate of decrease in the measured concept over time and positive values indicating increase over time. A significant negative covariance between the intercept and slope (describing the relationship between the starting point and the rate of change) is present in Confidence and Connection, indicating that higher initial levels of Confidence and Connection were related to a flatter slope in Confidence and Connection over time.

**TABLE 3:** Unconditional LGC model parameters and fit indices of the 5Cs over three time-points.

	Intercept			Slope		Model fit		
	*M*	Var	*r* (Intercept x Slope)	*M*	Var	CFI	RMSEA	SRMR
Connection	6.748***	0.267	-1.185*	-0.171**	0.063	0.994	0.041	0.009
Caring	6.457***	0.298	-0.057	-0.526**	0.179	0.998	0.023	0.007
Competence	3.371***	0.019	-0.004	0.018**	0.008	0.979	0.115	0.019
Confidence	4.275***	0.141	-0.267***	0.192**	0.065	1.000	0.000	0.003
Character	3.942***	0.017	-0.002	-0.020**	0.009	1.000	0.000	0.000

***p ≤ 0.001; **p ≤ 0.05; *p < 0.10.

#### Conditional LGC models

We present the findings of the conditional LGCMs, separately for each of the 5Cs, with three covariates (age, gender, school level), included as time-invariant predictors of the intercept and slope. This allows us to explain the variation of parameters between individuals.

**FIGURE 1:**
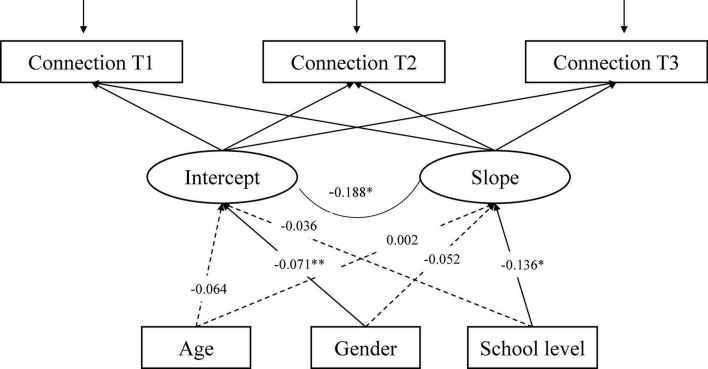
The conditional LGCM for Connection, measured at three time points (T1-T3), including three covariates (age, gender, and school level). The estimates are standardized coefficients. Solid lines represent significant paths and dash lines indicate non-significant paths. *p < 0.10, ^**^p < 0.01.

**FIGURE 2:**
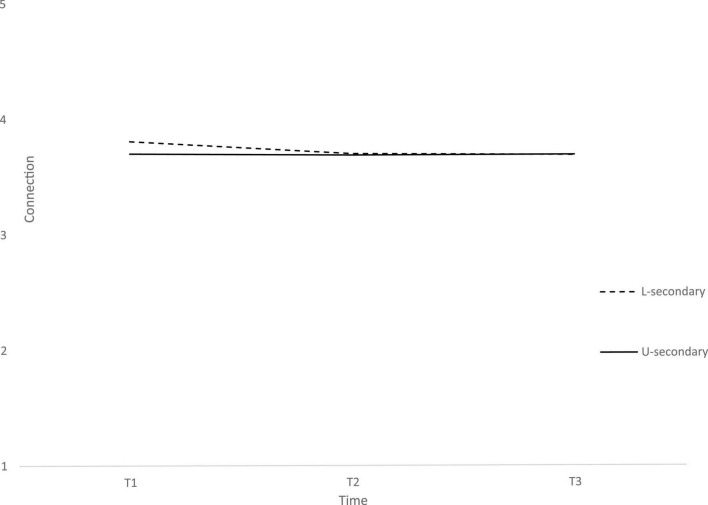
Connection at three-time points across two school levels: lower-secondary (L-secondary) and upper-secondary (U-secondary).

The LGCM for Connection (Figure 1), with the three time-invariant factors (age, gender, school level) as predictors of intercept and slope, showed an adequate fit to the data: CFI = 0.993, RMSEA = 0.032, 90% CI [0.000, 0.062], SRMR = 0.011. Gender is significantly associated with the intercept of Connection indicating that the initial levels of Connection vary across genders. Males report higher Connection compared to females. In addition, the school level is significantly associated with the rate of change. Thus, the rate of change in Connection differed across school levels (see details in Figure 2).

While the lower-secondary school students showed a decrease from T1 to T3, especially between T1 and T2, the upper-secondary school students remained relatively stable in Connection from T1 to T3.

**FIGURE 3:**
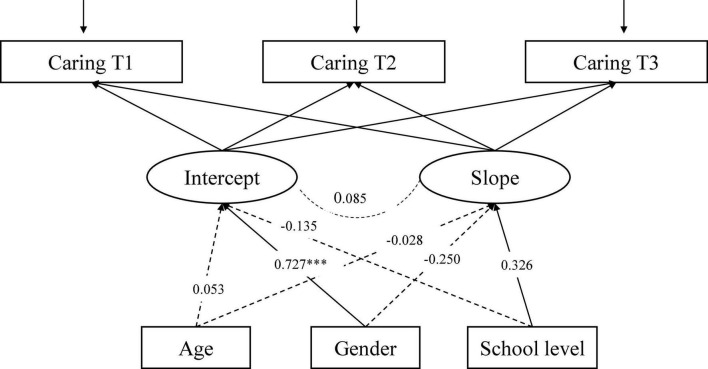
The conditional LGCM for Caring, measured at three time points (T1-T3), including three covariates (age, gender, and school level). The estimates are standardized coefficients. Solid lines represent significant paths and dash lines indicate non-significant paths ^***^p < 0.01.

**FIGURE 4:**
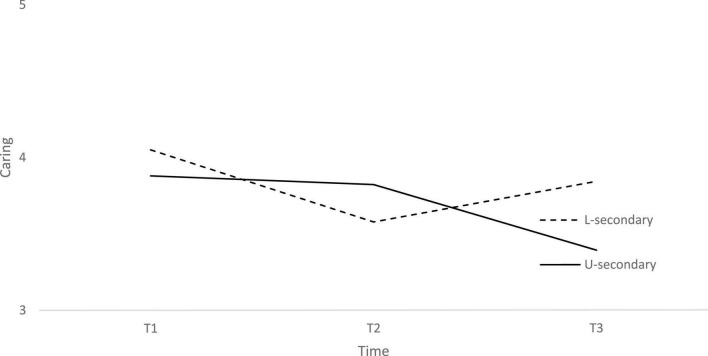
Caring at three time points across two school levels: lower-secondary (L-secondary) and upper-secondary (U-secondary).

The LGCM for Caring (Figure 3), with three time-invariant factors (age, gender, school type) as predictors of intercept and slope, showed an adequate fit to the data: CFI = 0.985, RMSEA = 0.053, 90% CI [0.030, 0.079], SRMR = 0.025. Gender is significantly associated with the intercept of Caring indicating that the initial levels of Caring vary between genders. Females report higher Caring compared to males. In addition, the school level is significantly associated with the rate of change. The rate of change in Caring differed across school levels (more in Figure 4).

In Figure 4, the rate of change differs between youth attending lower-secondary and youth attending upper-secondary school, the latter having a steeper drop from T2 to T3 and the former a steeper drop from T1 to T2, but a rise from T2 to T3.

**FIGURE 5:**
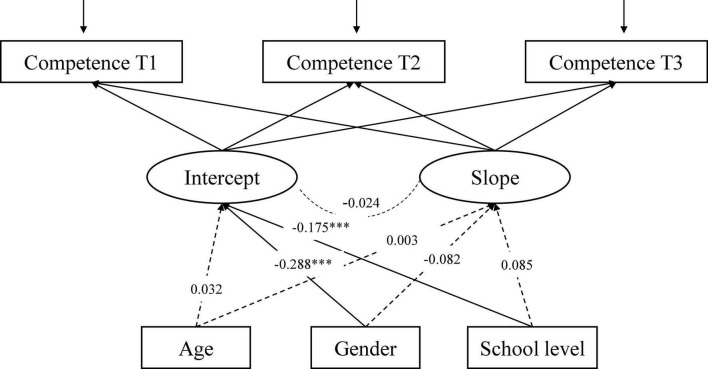
The conditional LGCM for Competence, measured at three time-points (T1-T3), including three covariates (age, gender, school level). The estimates are standardized coefficients. Solid lines represent significant paths and dash lines indicate non-significant paths; ***p < 0.001.

The LGCM for Competence (Figure 5), with three time-invariant factors (age, gender, school level) as predictors of intercept and slope, shows an adequate fit to the data: CFI = 0.988, RMSEA = 0.061, 90% CI [0.038, 0.087], SRMR = 0.021. Gender and school level were significant predictors of the intercept, which indicated significant differences in initial levels of Competence by gender and school level. Male gender and upper-secondary school students reported higher Competence.

**FIGURE 6:**
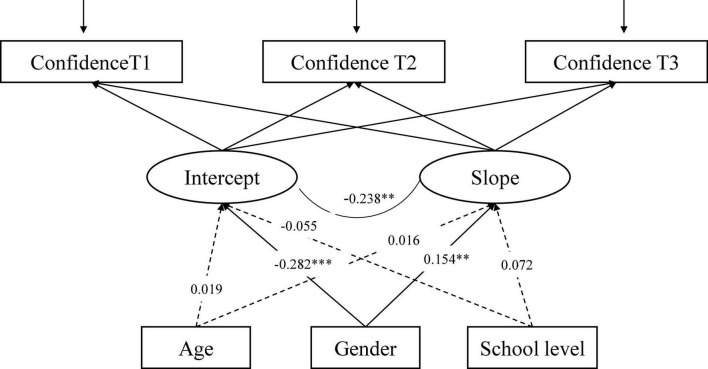
The conditional LGCM for Confidence, measured at three time points (T1-T3), including three covariates (age, gender, and school level). The estimates are standardized coefficients. Solid lines represent significant paths and dash lines indicate non-significant paths ^**^p < 0.05, ^***^p < 0.001.

**FIGURE 7:**
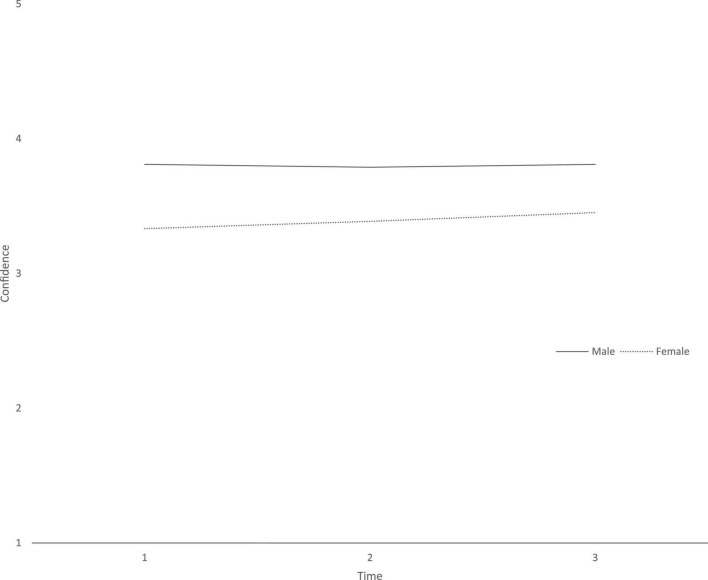
Confidence at three time points across genders.

The LGCM for Confidence (Figure 6), with three time-invariant factors (age, gender, school level) as predictors of intercept and slope, shows an adequate fit to the data: CFI = 0.998, RMSEA = 0.026, 90% CI [0.000, 0.055], SRMR = 0.007. Gender is significantly related to the slope and the intercept of Confidence, thus indicating significant differences in initial levels of Confidence by gender as well as the rate of change. The male gender is associated with higher Confidence and a slight decline from T1 to T2, while the female gender is associated with low Confidence and an increase from T1 to T3 (see Figure 7).

Males report higher Confidence at the initial level but a decrease from T2. While Females report lower initial levels and a steady increase from T1 to T3.

**FIGURE 8:**
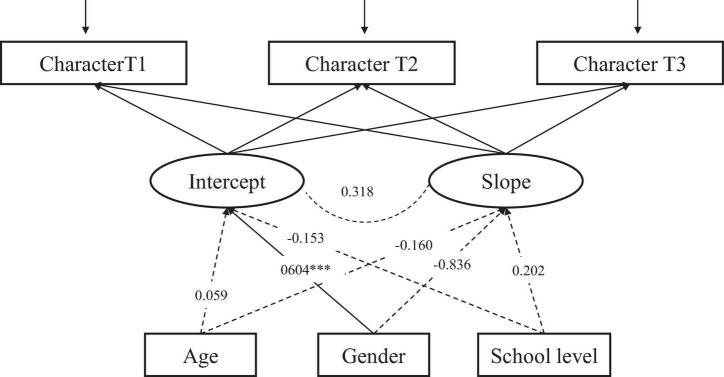
The conditional LGCM for Character, measured at three time points (T1-T3), including three covariates (age, gender, and school level). The estimates are standardized coefficients. Solid lines represent significant paths and dash lines indicate non-significant paths ***p < 0.001.

The LGCM for Character (Figure 8), with three time-invariant factors (age, gender, school level) as predictors of intercept and slope, showed good fit to the data: CFI = 0.994, RMSEA = 0.028, 90% CI [0.000, 0.057], SRMR = 0.012. Gender is significantly related to the intercept of Character, indicating that the initial levels vary between genders. Females report higher Character compared to males.

## Discussion

In the present study, we focused on indicators of positive youth developmental outcomes in one school year. We investigated longitudinal pathways for the 5Cs of Positive Youth Development in a representative sample of Slovenian youth during the COVID-19 pandemic. We used data from three-time points: the beginning of the school year, the middle of the school year, and the end of the school year. We followed all 5Cs, Competence, Confidence, Character, Caring, and Connection, from the beginning to the end of the school year and investigated their rate of change using Longitudinal Growth Curve Modeling (LGCM). Additionally, we were interested in knowing if the rate of change in the 5Cs was associated with age, gender, and school level.

After establishing measurement invariance across the three measurement points for all the 5Cs, we first focused on the unconditional LGCMs for all 5Cs, separately, to test the initial level of differences across the 5Cs and their rate of change across the three-time points. Even though US-based research (Lerner et al., 2017) showed stability in the 5Cs throughout adolescence, we hypothesized that the 5Cs would significantly change in one school year due to the characteristics of normative school processes on one hand (meso-changes in context) and the effects of the COVID-19 pandemic and its restrictions (macro change of context) on the other. In line with our expectations, the unconditional LGCMs for the 5Cs showed significant differences in intercept, indicating significant variations in the 5Cs of youth in Slovenia as well as significant differences in slope indicating significant change between measured time points during the school year. The findings reflect the basic premise of the PYD framework, that the 5Cs are outcomes of mutually reinforcing individual–context relationships (Lerner, 2007). Meso and macro changes, in our case, changes in the school context as well as the COVID-19 restrictions, are reflected in the change of the 5Cs. The simultaneous processes regarding changes in the school context and the pandemic progression in the development of youth make these impacts difficult to disentangle. The findings, however, still bring new insights into the possible impact of the COVID-19 pandemic, a macro-level change that influenced all youth.

The social distancing restrictions imposed due to the COVID-19 pandemic caused severe context change for all, especially youth (Avsec et al., 2021), therefore, we hypothesized a decrease in Connection throughout the school year. The present findings did indeed confirmed a significant decrease in Connection from the beginning to the end of the school year. Connection, as conceptualized in the PYD framework, is a combination of positive relationships across youth contexts: peers, friends, family, school, and community. The social distancing restrictions cut off all but family relationships for youth in our sample. By limiting the physical contact of adolescents to only their close family members, opportunities for youth to develop positive connections with peers, school, as well as a community, were restricted. As argued by Ettekal and Agans (2020), the caring relationship of youth with (non-parent) adult plays a significant role in fostering positive youth development. The need of youth for positive and caring relationships with adults during the pandemic was not met or was at least limited to virtual platforms.

As for covariates, gender was significantly associated with initial levels of Connection and school level to the rate of change. In contrast to the findings from international research (Conway et al., 2015; Årdal et al., 2018), males in our sample reported higher levels of Connection compared to females. Concerning the school level, the Connection of lower-secondary school students decreased from the beginning to the end of the school year, while the Connection of upper-secondary school students remained relatively stable in the same period. Age was not significantly associated with either the initial level or the rate of change. Thus, the differences in the rate of change across school levels cannot simply be attributed to the lower-secondary school students being younger. The explanations is likely to be found in the school level context. For instance, one of the differences in the school levels was the level of stability of the context, students in the lower-secondary schools had been together, in the same class, for the last 9 years, and by the end of the school year, they were ending one school level and entering the next level and new classes. Facing a transition period could explain why they felt less connected as the school year progressed. As stated by Bowers et al. (2010), as adolescents move to new learning environments, experience new social situations and autonomy become an important developmental goal, many adolescents may begin to doubt their academic and social abilities and, as well, may feel less connected to both parents, peers, and the larger ecological context. In contrast, the upper-secondary school students were a more heterogeneous sample consisting of several grades, not just the final one and this can result in greater stability in their case. Additional studies will be needed to probe into the association between the stability of peer groups and Connection. On the same note, it would be worth exploring which Connection context, peers, friends, family, school, or community was the one most affected by the COVID-19 pandemic.

As one needs social contacts to develop empathy and sympathy for others, we have also hypothesized a decrease in Caring. Our findings support our hypothesis as we found a significant decrease in Caring from the beginning to the end of the school year. Decrease in social contacts limits opportunities to practice empathy and sympathy. In addition, school as a context usually plays an important role in the development of Caring. Barr and Higgins-D’Alessandro (2009) showed longitudinal relationships between empathy and school culture. When school culture changes in a positive direction to become a caring community, change in perspective-taking follows together with a greater sense of connectedness and cooperation among students. As in Connection, we have also looked at covariates for Caring and established significant associations of gender with the initial levels of Caring and of school level with the rate of change. More specifically, and aligned with the literature (Conway et al., 2015; Årdal et al., 2018), females scored higher in Caring compared to males. School-level on the other hand was not associated with initial levels but with the rate of change. The rate of change differs between youth attending lower-secondary schools and youth attending upper-secondary schools. Lower-secondary school students had a steeper drop from the beginning to the middle of the school year.

The time from the beginning to the middle of the school year overlaps with the homeschooling restrictions, which shows how important direct physical contact with others is for developing and maintaining empathy and sympathy. As the school opened, the lower-secondary students showed a gradual increase from the middle of the school year to the end of the school year. In the sample of lower-secondary school students, we see the alignment of the development of their Caring with the changes in context, e.g., homeschooling. As upper secondary students were in homeschooling for a longer period with limited access even after the end of homeschooling, (for instance every second week in school and every second week at home), their level of Caring had not stabilized or increased by the end of the school year as it did in the lower-secondary school students. The findings in their case showed a significant decrease from the beginning to the end of the school year. We did have two contextual changes: the school context change in one school year and changes due to the COVID-19 pandemic. It is therefore hard to tell which context is contributing to the decrease. A clarification is needed in future studies that would also observe changes in the 5Cs in a school year without homeschooling and other COVID-19 restrictions. However, the alignment of our findings with the contextual changes imposed by the pandemic indicates a possible strong influence of the COVID-19 pandemic on the lives of youth.

Online teaching and learning implemented as a consequence of school closure was an additional significant change in the school context. Therefore, we have assumed that Competence and Confidence would decrease throughout the school year. On the contrary, our data showed a significant increase from the beginning to the end of the school year. We can therefore assume that the COVID-19 pandemic did not have a strong effect on the overall Competence and Confidence, and that youth despite the homeschooling and social restrictions managed to increase their Competence and Confidence throughout the school year. As for the covariates, aligned with previous research (Conway et al., 2015; Årdal et al., 2018), male gender reported higher Competence. Additionally, students enrolled in upper-secondary school reported higher Competence than students enrolled in lower-secondary school. Similar to Competence, Confidence showed a significant increase from the beginning to the end of the school year with males reporting higher Confidence at the initial level, again aligned with the literature (Gomez-Baya et al., 2019). Gender was also significantly associated with the rate of change in time. Males reported a drop from the beginning of the school year to the middle of the school year. As this was the period of stricter home-schooling rules and lockdown, we can assume male students had more difficulties with their self-esteem and self-efficacy in that period compared to females. They however increased their Confidence from the middle to the end of the school year. In the same period, female students reported lower initial levels and a steady increase from the beginning to the end of the school year.

For Character, we assumed it would be stable throughout the school year thinking that one school year would be too short to capture the developmental change in Character. Character tends to develop slowly throughout the adolescent years as adolescents tend to think progressively more about their role in the world, their identity, their meeting with diverse people and becoming more and more socially conscious (Park, 2004; Bowers et al., 2010). The present findings, however, showed a decrease from the beginning of the school year to the end of the school year and no significant covariates for the rate of change were detected. The decrease can again be attributed to the contextual changes of COVID-19 and social distancing measures together with home-schooling. Opportunities for actively interacting with peers and teachers have been found to promote or support the ability of students to respond appropriately to others’ situations, not just friends but other subgroups within the student population to which they may not belong (Barr and Higgins-D’Alessandro, 2009). Consistent with earlier research (Conway et al., 2015; Årdal et al., 2018) females reported higher Character compared to males.

In the unconditional LGM for Confidence and Connection, we also observed a significant interaction effect between initial levels of Confidence and Connections and their rate of change in one school year indicating that the rate of change is associated with the initial level. The more confident students felt at the beginning of the school year the more stable their Confidence was throughout the school year. Moreover, the more connected students felt at the beginning of the school year, the more stable, and less steep, was the change in Connection throughout the school year. These findings show how important the promotion of the 5Cs is to foster positive youth development. Even with the drop, due to the context change brought about by COVID-19, this drop was not so severe. We do however want to stress the severe impact that the homeschooling had on the positive youth development of youth in Slovenia reflected in the decrease in Connection, Caring, and Character. Home-schooling was especially long in Slovenia, and could have been an exaggerated measure, especially as it happened in the second wave of COVID-19, when it was clear that youth were not a risk group for prolonged recoveries after COVID-19. School is a primary socializing unit (Chhuon and Wallace, 2014) and a community that offers opportunities for students to develop their strengths. Thus, school has an obligation to provide a safe context and should be preserved as a basic human right for all children and adolescents regardless of the status of a pandemic. The decrease in Connection is even more alarming in adolescence where social contexts outside the family are fuel for normative social, emotional, and cognitive development (Scholte et al., 2001). Similarly, Ettekal and Agans (2020) indicated how the disruption of youth out-of-school time programs as essential ecological assets during the pandemic, might have had a major impact on youth developmental pathways. We also have to add that the possibilities of conflicts inside the close family context could be magnified by the COVID-19 lockdown (Bradbury-Jones and Isham, 2020).

### Implications for theory and practice

The present study is the first to investigate the changes of the 5Cs in one-school year during the COVID-19 pandemic in a representative youth sample, and as such brings important theoretical insights into the development of the 5Cs. The 5Cs are especially important due to them leading to the 6th C, that is Contribution, where adolescents are more likely to contribute to self, family, community, and civil society and less likely to be on a trajectory of risk and problem behaviors, such as substance abuse, delinquency, and depression (Phelps et al., 2009). In the light of theoretical insights, it is especially worrying to see the decrease in Connection, Caring and Character as these are the building blocks for the social cohesion and social progress of the society.

Our study, therefore, highlights the importance of the 5Cs and their promotion in times of pandemics as well as in more stable times. More specifically, from the significant interaction effects, we see how high levels of the Cs, as in the case of Connection and Confidence, provide a safety net for a more stable trajectory throughout the contextual changes triggered by the COVID-19 pandemic. In this regard, we highlight the need for PYD initiatives to empower youth for the challenges ahead of them as well as be able to provide instant and immediate support in times of crisis. Thus, support in the form of continuous professional development for teachers and other school staff is needed. A good example for such a support is the HAND in HAND program (Kozina et al., 2020) currently implemented in Slovenia and focusing on social and emotional as well as diversity awareness of teachers. These types of support could also be beneficial for effective teachers-parent collaboration with the aim to support positive youth development. The need to act and to learn from crisis is highlighted also in Lerner et al. (2021) review paper. Furthermore, the findings strongly support the importance of the context in nurturing and supporting positive youth development based on the observed alignment of a decrease in Connection, Caring, and Character with the ongoing COVID-19 restrictions. The decrease was more severe from the beginning to the middle of the school year, which coincided with the school closure. The same pattern was reflected in the male sample, regarding Confidence. Confidence dropped as homeschooling persisted and it recovered when schools reopened. This is an important empirical support for the significance of the school context.

### Limitations and future research directions

The study has several limitations, which should be considered in the interpretations of the present findings. First, it relies on the use of self-report measures and would in future studies benefit from inclusion of other measures, such as other reports (e.g., significant others), observations (e.g., in classroom), and sociometric (e.g., to analyze in more details the connections within peer groups). Further, additional analyses are needed on the effects of school context, e.g., focusing not only on the level but also on the school types in upper secondary level. Moreover, as the data collection took place in the non-normative context of homeschooling due to the COVID-19 restrictions, the present findings, although providing an interesting insight into youth development, is limited in their generalizations. In future studies, we would like to replicate the analyses in a non-COVID-19 context to compare the trajectories and have a better understanding of the individual-context relationship in one school year. Finally, the present findings also highlight the importance of gender and school level in several of the Cs. The latter triggers additional research questions regarding the effects of school levels and other factors related to the demographic characteristics of the school and students on the 5Cs trajectories in one school year. In future studies, additional possible interaction effects, for instance with migration status, can be examined.

## Conclusion

In their review of the PYD research in the last decades, Lerner et al. (2021) point out the need to add novelties and expansions in three ways: PYD as a theoretical construct, PYD as a framework for youth programs and PYD as a specific program. Our study brings new theoretical insights to PYD indicators and their development in the school context, from the beginning to the end of the school year, while at the same time, presenting the impact of the COVID-19 pandemic and its restrictions on positive youth development. As such, our findings provide information that can be used to develop and implement PYD programs in schools with the underlying goals of providing opportunities and nurturing contexts for positive development for all youth.

## Data availability statement

The raw data supporting the conclusions of this article will be made available by the authors, without undue reservation.

## Ethics statement

The studies involving human participants were reviewed and approved by Committee for Ethical Research at the Faculty of Arts of the University of Maribor. Written informed consent to participate in this study was provided by the participants’ legal guardian/next of kin.

## Author contributions

AK designed the study and collected the data. NW provided methodological support for data analyses. Both authors were involved in the critical review and manuscript revision, approved the final version of the manuscript, and involved in drafting and finalizing the manuscript.
